# Growth Analysis of Composite Entire and Meromorphic Functions in the Light of Their Relative Orders

**DOI:** 10.1155/2014/538327

**Published:** 2014-10-29

**Authors:** Sanjib Kumar Datta, Tanmay Biswas, Chinmay Biswas

**Affiliations:** ^1^Department of Mathematics, University of Kalyani, Kalyani, Nadia District, West Bengal 741235, India; ^2^Rajbari, Rabindra Pally, R. N. Tagore Road, Krishnagar, Nadia District, West Bengal 741101, India; ^3^Taraknagar Jamuna Sundari High School, Taraknagar, Hanskhali, Nadia District, West Bengal 741502, India

## Abstract

We study some comparative growth properties of composite entire and meromorphic functions on the basis of their relative orders (relative lower orders).

## 1. Introduction

Let *f* be meromorphic and *g* be an entire function defined in the open complex plane *ℂ*. The maximum modulus function corresponding to entire *g* is defined as *M*_*g*_(*r*) = max⁡{|*g*(*z*)| : |*z*| = *r*}. For meromorphic *f*, *M*_*f*_(*r*) cannot be defined as *f* is not analytic. In this situation one may define another function *T*_*f*_(*r*) known as Nevanlinna's characteristic function of *f*, playing the same role as maximum modulus function in the following manner:

(1) $$\begin{array}{c} T_{f}\left(r\right)=N_{f}\left(r\right)+m_{f}\left(r\right), \end{array}$$

where the function $N_{f}\left(r,a\right)({\bar{N}}_{f}\left(r,a\right))$ known as counting function of *a*-points (distinct *a*-points) of meromorphic *f* is defined as

(2) $$\begin{array}{c} N_{f}\left(r,a\right)=\overset{r}{\underset{0}{\int }}\frac{n_{f}\left(t,a\right)-n_{f}\left(0,a\right)}{t}dt+n_{f}\left(0,a\right)\log r,\\ \left({\bar{N}}_{f}\left(r,a\right)=\overset{r}{\underset{0}{\int }}\frac{{\overset{-}{n}}_{f}\left(t,a\right)-{\overset{-}{n}}_{f}\left(0,a\right)}{t}dt+{\bar{n}}_{f}\left(0,a\right)\log r\right). \end{array}$$

Moreover, we denote by $n_{f}\left(r,a\right)({\bar{n}}_{f}\left(r,a\right))$ the number of *a*-points (distinct *a*-points) of *f* in |*z*| ≤ *r* and an *∞*-point is a pole of *f*. In many occasions *N*_*f*_(*r*, *∞*) and ${\bar{N}}_{f}\left(r,\infty \right)$ are denoted by *N*_*f*_(*r*) and ${\bar{N}}_{f}\left(r\right)$, respectively.

The function *m*_*f*_(*r*, *∞*) alternatively denoted by *m*_*f*_(*r*) known as the proximity function of *f* is defined as follows:

(3) mf(r)=12π∫02πlog⁡+|f(reiθ)|dθ,where log⁡+x=max⁡(log⁡x,0) ∀x⩾0.

Also we may denote *m*(*r*, 1/(*f* − *a*)) by *m*_*f*_(*r*, *a*).

When *f* is an entire function, the Nevanlinna's characteristic function *T*_*f*_(*r*) of *f* is defined as

(4) $$\begin{array}{c} T_{f}\left(r\right)=m_{f}\left(r\right). \end{array}$$

Further, if *f* is a nonconstant entire function then *M*_*f*_(*r*) and *T*_*f*_(*r*) are both strictly increasing and continuous functions of *r*. Also their inverses *M*_*f*_^−1^(*r*) : (|*f*(0)|, *∞*) → (0, *∞*) and *T*_*f*_^−1^ : (*T*_*f*_(0), *∞*) → (0, *∞*) exist, respectively, and are such that lim⁡_*s*→*∞*_⁡*M*_*g*_^−1^(*s*) = *∞* and lim⁡_*s*→*∞*_⁡*T*_*f*_^−1^(*s*) = *∞*.

In this connection we just recall the following definition which is relevant.

**Definition 1 (see [1]).**

A nonconstant entire function *f* is said to have the Property (A) if, for any *σ* > 1 and for all sufficiently large *r*, [*M*_*f*_(*r*)]^2^ ≤ *M*_*f*_(*r* ^*σ*^) holds. For examples of functions with or without the Property (A), one may see [1].

However, for any two entire functions *f* and *g*, the ratio *M*_*f*_(*r*)/*M*_*g*_(*r*) as *r* → *∞* is called the growth of *f* with respect to *g* in terms of their maximum moduli. Similarly, when *f* and *g* are both meromorphic functions, the ratio *T*_*f*_(*r*)/*T*_*g*_(*r*) as *r* → *∞* is called the growth of *f* with respect to *g* in terms of their Nevanlinna's characteristic functions. The notion of the growth indicators such as* order* and* lower order* of entire or meromorphic functions which are generally used in computational purpose is defined in terms of their growth with respect to the* exponential* function as the following.

**Definition 2 .**

The order *ρ*_*f*_ (the lower order *λ*_*f*_) of an entire function *f* is defined as

(5) $$\begin{array}{c} \rho_{f}=\underset{r\to \infty }{\mathrm{limsup}}\frac{\log\log M_{f}\left(r\right)}{\log\log M_{\exp z}\left(r\right)}=\underset{r\to \infty }{\mathrm{limsup}}\frac{\log^{\left[2\right]}M_{f}\left(r\right)}{\log r}\\ \left(\lambda_{f}=\underset{r\to \infty }{\mathrm{liminf}}\frac{\log\log M_{f}\left(r\right)}{\log\log M_{\exp z}\left(r\right)}=\underset{r\to \infty }{\mathrm{liminf}}\frac{\log^{\left[2\right]}M_{f}\left(r\right)}{\log r}\right), \end{array}$$

where log⁡^[*k*]^⁡*x* = log⁡⁡(log⁡^[*k*−1]^⁡*x*) for *k* = 1,2, 3,… and log⁡^[0]^⁡*x* = *x*. Further, if *f* is a meromorphic function one can easily verify that

(6) ρf=limsup⁡r→∞log⁡Tf(r)log⁡Texp⁡z(r)=limsup⁡r→∞log⁡Tf(r)log⁡(r/π)=limsup⁡r→∞log⁡Tf(r)log⁡r+O(1),(λf=liminf⁡r→∞log⁡Tf(r)log⁡Texp⁡z(r)=liminf⁡r→∞log⁡Tf(r)log⁡(r/π)  =liminf⁡r→∞log⁡Tf(r)log⁡r+O(1)).

Bernal [1, 2] introduced the definition of relative order of an entire function *f* with respect to another entire function *g*, denoted by *ρ*_*g*_(*f*) to avoid comparing growth just with exp⁡*z* as follows:

(7) ρg(f) =inf⁡{μ>0:Mf(r)<Mg(rμ) ∀r>r0(μ)>0} =limsup⁡r→∞log⁡⁡Mg−1Mf(r)log⁡⁡r.

The definition coincides with the classical one [3] if *g*(*z*) = exp⁡*z*.

Similarly, one can define the relative lower order of an entire function *f* with respect to another entire function *g* denoted by *λ*_*g*_(*f*) as follows:

(8) $$\begin{array}{c} \lambda_{g}\left(f\right)=\underset{r\to \infty }{\mathrm{liminf}}\frac{\log M_{g}^{-1}M_{f}\left(r\right)}{\log r}. \end{array}$$

Extending this notion, Lahiri and Banerjee [4] introduced the definition of relative order of a meromorphic function with respect to an entire function in the following way.

**Definition 3 (see [4]).**

Let *f* be any meromorphic function and *g* be any entire function. The relative order of *f* with respect to *g* is defined as

(9) ρg(f) =inf⁡⁡{μ>0:Tf(r)<Tg(rμ) for all sufficiently large  r} =limsup⁡r→∞log⁡Tg−1Tf(r)log⁡r.

Likewise, one can define the relative lower order of a meromorphic function *f* with respect to an entire function *g* denoted by *λ*_*g*_(*f*) as follows:

(10) $$\begin{array}{c} \lambda_{g}\left(f\right)=\underset{r\to \infty }{\mathrm{liminf}}\frac{\log T_{g}^{-1}T_{f}\left(r\right)}{\log r}. \end{array}$$

It is known (cf. [4]) that if *g*(*z*) = exp⁡*z* then Definition 3 coincides with the classical definition of the order of a meromorphic function *f*.

For entire and meromorphic functions, the notions of their growth indicators such as* order* and* lower order* are classical in complex analysis and during the past decades, several researchers have already been exploring their studies in the area of comparative growth properties of composite entire and meromorphic functions in different directions using the classical growth indicators. But at that time, the concepts of* relative orders* and* relative lower orders* of entire and meromorphic functions and their technical advantages of not comparing with the growths of exp⁡*z* are not at all known to the researchers of this area. Therefore the studies of the growths of composite entire and meromorphic functions in the light of their* relative orders* and* relative lower orders* are the prime concern of this paper. In fact some light has already been thrown on such type of works by Datta et al. [5]. We do not explain the standard definitions and notations of the theory of entire and meromorphic functions as those are available in [6, 7].

## 2. Lemmas

In this section we present some lemmas which will be needed in the sequel.

**Lemma 1 (see [8]).**

Let *f* be meromorphic and *g* be entire; then, for all sufficiently large values of *r*,

(11) $$\begin{array}{c} T_{f\circ g}\left(r\right)⩽\left\{1+o\left(1\right)\right\}\frac{T_{g}\left(r\right)}{\log M_{g}\left(r\right)}T_{f}\left(M_{g}\left(r\right)\right). \end{array}$$

**Lemma 2 (see [9]).**

Let *f* be meromorphic and *g* be entire and suppose that 0 < *μ* < *ρ*_*g*_ ≤ *∞*. Then, for a sequence of values of *r* tending to infinity,

(12) $$\begin{array}{c} T_{f\circ g}\left(r\right)\geq T_{f}\left(\exp\left(r^{\mu }\right)\right). \end{array}$$

**Lemma 3 (see [10]).**

Let *f* be meromorphic and *g* be entire such that 0 < *ρ*_*g*_ < *∞* and 0 < *λ*_*f*_. Then, for a sequence of values of *r* tending to infinity,

(13) $$\begin{array}{c} T_{f\circ g}\left(r\right)>T_{g}\left(\exp\left(r^{\mu }\right)\right), \end{array}$$

where 0 < *μ* < *ρ*_*g*_.

**Lemma 4 (see [11]).**

Let *f* be an entire function which satisfies the Property (A), *β* > 0, *δ* > 1, and *α* > 2. Then

(14) $$\begin{array}{c} \beta T_{f}\left(r\right)<T_{f}\left(\alpha r^{\delta }\right). \end{array}$$

## 3. Theorems

In this section we present the main results of the paper.

**Theorem 1 .**

Let *f* be a meromorphic function and *h* be an entire function with 0 < *λ*_*h*_(*f*) ≤ *ρ*_*h*_(*f*) < *∞* and let *g* be an entire function with finite order. If *h* satisfies the Property (A), then, for every positive constant *μ* and each *α* ∈ (−*∞*, *∞*),

(15) $$\begin{array}{c} \underset{r\to \infty }{\lim}\frac{{\left\{\log T_{h}^{-1}T_{f\circ g}\left(r\right)\right\}}^{1+\alpha }}{\log T_{h}^{-1}T_{f}\left(\exp r^{\mu }\right)}=0,\;where\;\;\mu >\left(1+\alpha \right)\rho_{g}. \end{array}$$

**Proof**

Let us suppose that *β* > 2 and *δ* > 1. If 1 + *α* ≤ 0, then the theorem is obvious. We consider 1 + *α* > 0.

Since *T*_*h*_^−1^(*r*) is an increasing function of *r*, it follows from Lemma 1, Lemma 4, and the inequality *T*_*g*_(*r*) ≤ log⁡*M*_*g*_(*r*)  {cf. [6]} for all sufficiently large values of *r* that

(16) Th−1Tf∘g(r)⩽Th−1[{1+o(1)}Tf(Mg(r))],that  is, Th−1Tf∘g(r)⩽β[Th−1Tf(Mg(r))]δ,that  is, log⁡Th−1Tf∘g(r)⩽δlog⁡Th−1Tf(Mg(r))+O(1),that  is, log⁡Th−1Tf∘g(r)⩽δ(ρh(f)+ɛ)rρg+ɛ+O(1).

Again for all sufficiently large values of *r* we get that

(17) $$\begin{array}{c} \log T_{h}^{-1}T_{f}\left(\exp r^{\mu }\right)\geq \left(\lambda_{h}\left(f\right)-ɛ\right)r^{\mu }. \end{array}$$

Hence for all sufficiently large values of *r* we obtain from (16) and (17) that

(18) {log⁡Th−1Tf∘g(r)}1+αlog⁡Th−1Tf(exp⁡rμ) ≤[δ(ρh(f)+ɛ)rρg+ɛ+O(1)]1+α(λh(f)−ɛ)rμ,

where we choose 0 < *ɛ* < min⁡{*λ*_*h*_(*f*), (*μ*/(1 + *α*)) − *ρ*_*g*_}.

So from (18) we obtain that

(19) $$\begin{array}{c} \underset{r\to \infty }{\lim}\frac{{\left\{\log T_{h}^{-1}T_{f\circ g}\left(r\right)\right\}}^{1+\alpha }}{\log T_{h}^{-1}T_{f}\left(\exp r^{\mu }\right)}=0. \end{array}$$

This proves the theorem.

**Remark 2 .**

In Theorem 1 if we take the condition 0 < *ρ*_*h*_(*f*) < *∞* instead of 0 < *λ*_*h*_(*f*) ≤ *ρ*_*h*_(*f*) < *∞*, the theorem remains true with “*limit inferior*” in place of “*limit*.”

In view of Theorem 1 the following theorem can be carried out.

**Theorem 3 .**

Let *f* be a meromorphic function and let *g*, *h* be any two entire functions where *g* is of finite order and *λ*_*h*_(*g*) > 0, *ρ*_*h*_(*f*) < *∞*. If *h* satisfies the Property (A), then, for every positive constant *μ* and each *α* ∈ (−*∞*, *∞*),

(20) $$\begin{array}{c} \underset{r\to \infty }{\lim}\frac{{\left\{\log T_{h}^{-1}T_{f\circ g}\left(r\right)\right\}}^{1+\alpha }}{\log T_{h}^{-1}T_{g}\left(\exp r^{\mu }\right)}=0,\;where\;\;\mu >\left(1+\alpha \right)\rho_{g}. \end{array}$$

The proof is omitted.

**Remark 4 .**

In Theorem 3 if we take the condition *ρ*_*h*_(*g*) > 0 instead of *λ*_*h*_(*g*) > 0, the theorem remains true with “*limit*” replaced by “*limit inferior*”.

**Theorem 5 .**

Let *f* be a meromorphic function and let *g*, *h* be any two entire functions such that 0 < *λ*_*h*_(*f*) ≤ *ρ*_*h*_(*f*) < *∞* and *λ*_*g*_ < *μ* < *∞*. Also suppose that *h* satisfies the Property (A). Then, for a sequence of values of *r* tending to infinity,

(21) $$\begin{array}{c} T_{h}^{-1}T_{f\circ g}\left(r\right)<T_{h}^{-1}T_{f}\left(\exp r^{\mu }\right). \end{array}$$

**Proof**

Let us consider *δ* > 1. Since *T*_*h*_^−1^(*r*) is an increasing function of *r*, it follows from Lemma 1 that, for a sequence of values of *r* tending to infinity,

(22) $$\begin{array}{c} \log T_{h}^{-1}T_{f\circ g}\left(r\right)⩽\delta \left(\rho_{h}\left(f\right)+ɛ\right)r^{\lambda_{g}+ɛ}+O\left(1\right). \end{array}$$

Now from (17) and (22), it follows for a sequence of values of *r* tending to infinity that

(23) $$\begin{array}{c} \frac{\log T_{h}^{-1}T_{f}\left(\exp r^{\mu }\right)}{\log T_{h}^{-1}T_{f\circ g}\left(r\right)}\geq \frac{\left(\lambda_{h}\left(f\right)-ɛ\right)r^{\mu }}{\delta \left(\rho_{h}\left(f\right)+ɛ\right)r^{\lambda_{g}+ɛ}+O\left(1\right)}. \end{array}$$

As *λ*_*g*_ < *μ* we can choose *ɛ* (>0) in such a way that

(24) $$\begin{array}{c} \lambda_{g}+ɛ<\mu <\rho_{g}. \end{array}$$

Thus from (23) and (24) we obtain that

(25) $$\begin{array}{c} \underset{r\to \infty }{\mathrm{limsup}}\frac{\log T_{h}^{-1}T_{f}\left(\exp r^{\mu }\right)}{\log T_{h}^{-1}T_{f\circ g}\left(r\right)}=\infty . \end{array}$$

Now from (25), we obtain for a sequence of values of *r* tending to infinity and also for *K* > 1

(26) $$\begin{array}{c} T_{h}^{-1}T_{f}\left(\exp r^{\mu }\right)>T_{h}^{-1}T_{f\circ g}\left(r\right). \end{array}$$

Thus the theorem follows.

In the line of Theorem 5, we may state the following theorem without its proof.

**Theorem 6 .**

Let *g* and *h* be any two entire functions with *λ*_*h*_(*g*) > 0 and let *f* be a meromorphic function with finite relative order with respect to *h*. Also suppose that *λ*_*g*_ < *μ* < *∞* and *h* satisfies the Property (A). Then, for a sequence of values of *r* tending to infinity,

(27) $$\begin{array}{c} T_{h}^{-1}T_{f\circ g}\left(r\right)<T_{h}^{-1}T_{g}\left(\exp r^{\mu }\right). \end{array}$$

As an application of Theorem 5 and Lemma 2, we may state the following theorem.

**Theorem 7 .**

Let *f* be a meromorphic function and let *g*, *h* be any two entire functions such that 0 < *λ*_*h*_(*f*) ≤ *ρ*_*h*_(*f*) < *∞* and *λ*_*g*_ < *μ* < *ρ*_*g*_. If *h* satisfies the Property (A), then

(28) $$\begin{array}{c} \underset{r\to \infty }{\mathrm{liminf}}\frac{T_{h}^{-1}T_{f\circ g}\left(r\right)}{T_{h}^{-1}T_{f}\left(\exp r^{\mu }\right)}\leq 1\leq \underset{r\to \infty }{\mathrm{limsup}}\frac{T_{h}^{-1}T_{f\circ g}\left(r\right)}{T_{h}^{-1}T_{f}\left(\exp r^{\mu }\right)}. \end{array}$$

The proof is omitted.

Similary in view of Theorem 6 and Lemma 3, the following theorem can be carried out.

**Theorem 8 .**

Let *f* be a meromorphic function and let *g*, *h* be any two entire functions with 0 < *λ*_*h*_(*f*) ≤ *ρ*_*h*_(*f*) < *∞*, 0 < *λ*_*h*_(*g*) ≤ *ρ*_*h*_(*g*) < *∞*, and 0 < *λ*_*g*_ < *μ* < *ρ*_*g*_ < *∞*. Moreover *h* satisfies the Property (A). Then

(29) $$\begin{array}{c} \underset{r\to \infty }{\mathrm{liminf}}\frac{T_{h}^{-1}T_{f\circ g}\left(r\right)}{T_{h}^{-1}T_{g}\left(\exp r^{\mu }\right)}\leq 1\leq \underset{r\to \infty }{\mathrm{limsup}}\frac{T_{h}^{-1}T_{f\circ g}\left(r\right)}{T_{h}^{-1}T_{g}\left(\exp r^{\mu }\right)}. \end{array}$$

The proof is omitted.

**Theorem 9 .**

Let *f* be a meromorphic function and let *h*, *g* be any two entire functions with *λ*_*h*_(*f*) > 0 and 0 < *ρ*_*h*_(*g*) < *∞*. Then

(30) $$\begin{array}{c} \underset{r\to \infty }{\mathrm{limsup}}\frac{\log T_{h}^{-1}T_{f\circ g}\left(r\right)}{\log T_{h}^{-1}T_{g}\left(\exp r^{\mu }\right)}=\infty , \end{array}$$

where 0 < *μ* < *ρ*_*g*_.

**Proof**

Let 0 < *μ* < *μ* ^/^ < *ρ*_*g*_. As *T*_*h*_^−1^(*r*) is an increasing function of *r*, it follows from Lemma 2 for a sequence of values of *r* tending to infinity that

(31) log⁡⁡Th−1Tf∘g(r)≥log⁡⁡Th−1Tf(exp⁡(rμ/)),that  is, log⁡⁡Th−1Tf∘g(r)≥(λh(f)−ɛ)rμ/.

Again for all sufficiently large values of *r* we get that

(32) $$\begin{array}{c} \log T_{h}^{-1}T_{g}\left(\exp r^{\mu }\right)\leq \left(\rho_{h}\left(g\right)+ɛ\right)r^{\mu }. \end{array}$$

So combining (31) and (32), we obtain for a sequence of values of *r* tending to infinity that

(33) $$\begin{array}{c} \frac{\log T_{h}^{-1}T_{f\circ g}\left(r\right)}{\log T_{h}^{-1}T_{g}\left(\exp r^{\mu }\right)}\geq \frac{\left(\lambda_{h}\left(f\right)-ɛ\right)r^{\mu^{/}}}{\left(\rho_{h}\left(g\right)+ɛ\right)r^{\mu }}. \end{array}$$

Since *μ* < *μ* ^/^, it follows from (33) that

(34) $$\begin{array}{c} \underset{r\to \infty }{\mathrm{limsup}}\frac{\log T_{h}^{-1}T_{f\circ g}\left(r\right)}{\log T_{h}^{-1}T_{g}\left(\exp r^{\mu }\right)}=\infty . \end{array}$$

Hence the theorem follows.

**Corollary 10 .**

Under the assumptions of Theorem 9,

(35) $$\begin{array}{c} \underset{r\to \infty }{\mathrm{limsup}}\frac{T_{h}^{-1}T_{f\circ g}\left(r\right)}{T_{h}^{-1}T_{g}\left(\exp r^{\mu }\right)}=\infty ,\;0<\mu <\rho_{g}. \end{array}$$

**Proof**

In view of Theorem 9, we get for a sequence of values of *r* tending to infinity that

(36) log⁡Th−1Tf∘g(r)≥Klog⁡⁡Th−1Tg(exp⁡rμ), for  K>1,that  is, Th−1Tf∘g(r)≥log⁡{Th−1Tg(exp⁡rμ)}K,

from which the corollary follows.

**Theorem 11 .**

Let *f* be a meromorphic function and let *h*, *g* be any two entire functions such that (i) 0 < *ρ*_*h*_(*g*) < *∞*, (ii) *λ*_*h*_(*f*) > 0, and (iii) *λ*_*h*_(*f*∘*g*) > 0. Then

(37) $$\begin{array}{c} \underset{r\to \infty }{\mathrm{limsup}}\frac{{\left[\log T_{h}^{-1}T_{f\circ g}\left(r\right)\right]}^{2}}{\left\{\log T_{h}^{-1}T_{g}\left(\exp r^{\mu }\right)\right\}\cdot \left\{\log T_{h}^{-1}T_{g}\left(r\right)\right\}}=\infty , \end{array}$$

where 0 < *μ* < *ρ*_*g*_.

**Proof**

From the definition of relative order and relative lower order, we obtain for arbitrary positive *ɛ* and for all sufficiently large values of *r* that

(38) $$\begin{array}{c} \log T_{h}^{-1}T_{f\circ g}\left(r\right)\geq \left(\lambda_{h}\left(f\circ g\right)-ɛ\right)\log r,\\ \log T_{h}^{-1}T_{g}\left(r\right)\leq \left(\rho_{h}\left(g\right)+ɛ\right)\log r. \end{array}$$

Therefore, from (38), it follows for all sufficiently large values of *r* that

(39) log⁡Th−1Tf∘g(r)log⁡Th−1Tg(r)≥(λh(f∘g)−ɛ)log⁡r(ρh(g)+ɛ)log⁡r,that  is, liminf⁡r→∞log⁡Th−1Tf∘g(r)log⁡Th−1Tg(r)≥λh(f∘g)ρh(g).

Thus the theorem follows from (34) and (39).

Similarly, one may state the following theorems and corollary without their proofs as those can be carried out in the line of Theorems 9 and 11 and Corollary 10, respectively.

**Theorem 12 .**

Let *f* be a meromorphic function and *h* be an entire function with 0 < *λ*_*h*_(*f*) ≤ *ρ*_*h*_(*f*) < *∞*. Then, for any entire function *g*,

(40) $$\begin{array}{c} \underset{r\to \infty }{\mathrm{limsup}}\frac{\log T_{h}^{-1}T_{f\circ g}\left(r\right)}{\log T_{h}^{-1}T_{f}\left(\exp r^{\mu }\right)}=\infty , \end{array}$$

where 0 < *μ* < *ρ*_*g*_.

**Theorem 13 .**

Let *f* be a meromorphic function and let *h*, *g* be any two entire functions such that (i) 0 < *λ*_*h*_(*f*) ≤ *ρ*_*h*_(*f*) < *∞* and (ii) *λ*_*h*_(*f*∘*g*) > 0. Then

(41) $$\begin{array}{c} \underset{r\to \infty }{\mathrm{limsup}}\frac{{\left[\log T_{h}^{-1}T_{f\circ g}\left(r\right)\right]}^{2}}{\left\{\log T_{h}^{-1}T_{f}\left(\exp r^{\mu }\right)\right\}\cdot \left\{\log T_{h}^{-1}T_{f}\left(r\right)\right\}}=\infty , \end{array}$$

where 0 < *μ* < *ρ*_*g*_.

**Corollary 14 .**

Under the assumptions of Theorem 12,

(42) $$\begin{array}{c} \underset{r\to \infty }{\mathrm{limsup}}\frac{T_{h}^{-1}T_{f\circ g}\left(r\right)}{T_{h}^{-1}T_{f}\left(\exp r^{\mu }\right)}=\infty ,\;0<\mu <\rho_{g}. \end{array}$$

**Theorem 15 .**

Let *f* be a meromorphic function and let *h* be an entire function with 0 < *λ*_*h*_(*f*) ≤ *ρ*_*h*_(*f*) < *∞*. Then, for any entire function *g*,

(43) $$\begin{array}{c} \underset{r\to \infty }{\mathrm{limsup}}\frac{\log^{\left[2\right]}T_{h}^{-1}T_{f\circ g}\left(\exp r^{B}\right)}{\log^{\left[2\right]}T_{h}^{-1}T_{f}\left(\exp r^{\mu }\right)}=\infty , \end{array}$$

where 0 < *μ* < *ρ*_*g*_ and *B* > 0.

**Proof**

Let 0 < *μ* ^/^ < *ρ*_*g*_. As *T*_*h*_^−1^(*r*) is an increasing function of *r*, it follows from (31) for a sequence of values of *r* tending to infinity that

(44) $$\begin{array}{c} \log^{\left[2\right]}T_{h}^{-1}T_{f\circ g}\left(r\right)\geq O\left(1\right)+\mu^{/}\log r. \end{array}$$

So for a sequence of values of *r* tending to infinity we get from above that

(45) $$\begin{array}{c} \log^{\left[2\right]}T_{h}^{-1}T_{f\circ g}\left(\exp r^{B}\right)\geq O\left(1\right)+\mu^{/}r^{B}. \end{array}$$

Again we have for all sufficiently large values of *r* that

(46) $$\begin{array}{c} \log^{\left[2\right]}T_{h}^{-1}T_{f}\left(\exp r^{\mu }\right)\leq O\left(1\right)+\mu \log r. \end{array}$$

Now combining (45) and (46), we obtain for a sequence of values of *r* tending to infinity that

(47) $$\begin{array}{c} \frac{\log^{\left[2\right]}T_{h}^{-1}T_{f\circ g}\left(\exp r^{B}\right)}{\log^{\left[2\right]}T_{h}^{-1}T_{f}\left(\exp r^{\mu }\right)}\geq \frac{O\left(1\right)+\mu^{/}r^{B}}{O\left(1\right)+\mu \log r}, \end{array}$$

from which the theorem follows.

In view of Theorem 15 the following theorem can be carried out.

**Theorem 16 .**

Let *f* be a meromorphic function and let *h*, *g* be any two entire functions with *λ*_*h*_(*f*) > 0 and 0 < *ρ*_*h*_(*g*) < *∞*. Then

(48) $$\begin{array}{c} \underset{r\to \infty }{\mathrm{limsup}}\frac{\log^{\left[2\right]}T_{h}^{-1}T_{f\circ g}\left(\exp r^{B}\right)}{\log^{\left[2\right]}T_{h}^{-1}T_{g}\left(\exp r^{\mu }\right)}=\infty , \end{array}$$

where 0 < *μ* < *ρ*_*g*_ and *B* > 0.

The proof is omitted.

**Theorem 17 .**

Let *l* be an entire function satisfying the Property (A) and let *h* be a meromorphic function such that *λ*_*l*_(*h*) > 0. Also let *g* and *k* be any two entire functions with finite nonzero order such that *ρ*_*g*_ < *ρ*_*k*_. Then, for every meromorphic function *f* with 0 < *ρ*_*l*_(*f*) < *∞*,

(49) $$\begin{array}{c} \underset{r\to \infty }{\mathrm{limsup}}\frac{\log T_{l}^{-1}T_{h\circ k}\left(r\right)}{\log T_{l}^{-1}T_{f\circ g}\left(r\right)+\log T_{l}^{-1}T_{f}\left(r\right)}=\infty . \end{array}$$

**Proof**

Since *ρ*_*g*_ < *ρ*_*k*_, we can choose *ɛ* (>0) in such a way that

(50) $$\begin{array}{c} \rho_{g}+ɛ<\mu <\rho_{k}-ɛ. \end{array}$$

As *T*_*l*_^−1^(*r*) is an increasing function of *r*, it follows from Lemma 2 for a sequence of values of *r* tending to infinity that

(51) log⁡Tl−1Th∘k(r)≥log⁡Tl−1Th(exp⁡rμ),      where  0<μ<ρk≤∞; that  is, log⁡Tl−1Th∘k(r)≥(λl(h)−ɛ)rμ.

Now from the definition of relative order of *f* with respect to *l* we have for arbitrary positive *ɛ* and for all sufficiently large values of *r* that

(52) $$\begin{array}{c} \log T_{l}^{-1}T_{f}\left(r\right)\leq \left(\rho_{l}\left(f\right)+ɛ\right)\log r. \end{array}$$

Now for any *δ* > 1, we get from (16), (51), (52), and in view of (50) for a sequence of values of *r* tending to infinity that

(53) log⁡Tl−1Th∘k(r)log⁡Tl−1Tf∘g(r)+log⁡Tl−1Tf(r) ≥(λl(h)−ɛ)rμδ(ρh(f)+ɛ)rρg+ɛ+(ρl(f)+ɛ)log⁡r+O(1), that  is, log⁡Tl−1Th∘k(r)log⁡Tl−1Tf∘g(r)+log⁡Tl−1Tf(r)=∞,

which proves the theorem.

In the line of Theorem 17 the following theorem can be carried out.

**Theorem 18 .**

Let *l* be an entire function satisfying the Property (A) and let *h* be a meromorphic function such that *λ*_*l*_(*h*) > 0. Also let *g* and *k* be any two entire functions with finite nonzero order and also *ρ*_*g*_ < *ρ*_*k*_. Then, for every meromorphic function *f* with 0 < *ρ*_*l*_(*f*) < *∞*,

(54) $$\begin{array}{c} \underset{r\to \infty }{\mathrm{limsup}}\frac{\log T_{l}^{-1}T_{h\circ k}\left(r\right)}{\log T_{l}^{-1}T_{f\circ g}\left(r\right)+\log T_{l}^{-1}T_{g}\left(r\right)}=\infty . \end{array}$$

## 4. Conclusion

Actually this paper deals with the extension of the works on the growth properties of composite entire and meromorphic functions on the basis of their* relative orders* and* relative lower orders*. These theories can also be modified by the treatment of the notions of* generalized relative orders* (*generalized relative lower orders*) and (*p*, *q*)th* relative orders* ((*p*, *q*)th* relative lower orders*). Moreover, some extensions of the same type may be done in the light of slowly changing functions.
